# Korean adolescents’ experiences studying abroad and subsequent readjustment to life after returning

**DOI:** 10.3389/fpsyg.2024.1499557

**Published:** 2024-12-09

**Authors:** Ji-yeon Lee, Dong Hun Lee

**Affiliations:** ^1^Department of Counseling Psychology, Graduate School of Education, Hankuk University of Foreign Studies, Seoul, Republic of Korea; ^2^Department of Education, Traumatic Stress Center, Sungkyunkwan University, Seoul, Republic of Korea

**Keywords:** returning Korean adolescents, studying abroad, re-adjustment, qualitative study, phenomenology

## Abstract

**Introduction:**

This qualitative study explored the socio-cultural adjustment and re-adjustment experiences of South Korean adolescents who studied in English-speaking countries and later returned to South Korea.

**Methods:**

The study interviewed 12 adolescents (6 males, 6 females) aged 12–16 who studied in countries such as the U.S., Canada, England, New Zealand, and Australia.

**Results:**

The findings highlight the students’ adjustment processes abroad, including the initial “honeymoon” phase, followed by crises like language barriers, homesickness, and difficulties in relationships with peers and teachers. Upon returning to Korea, students faced challenges with academic performance, stricter school regulations, and social reintegration, often exacerbated by biases toward those who studied abroad.

**Discussion:**

The study underscores the importance of support systems during both phases and highlights the need for educational interventions that can ease the transition both abroad and at home. This research contributes to understanding the complexities of cross-cultural adjustment and re-entry, especially in the context of adolescents’ identity and belonging, suggesting sustainable education system to support students’ both abroad and returning process.

## Introduction

International student issues are an important topic for scholars who are interested in cross-cultural adjustment issues, as there is an increasing number of students who decide to study abroad worldwide. Most of the research on this topic has focused on difficulties related to international students’ adjustment to the host culture (Chapdelaine and Alexitch, 2004; Lee, 2010; Lee et al., 2004; Ma et al., 2020; Sandhu, 1994; Sumer et al., 2008; Yoon and Portman, 2004), their academic performance (Reynolds and Constantine, 2007; Toyokawa and Toyokawa, 2002; Yeh and Inose, 2003), and the acculturation process (Bui et al., 2021; Nilsson et al., 2008; Poyrazil et al., 2004; Smith and Khawaja, 2011; Lee and Ciftci, 2014), but there has been little attention to re-adjustment issues (e.g., socio-cultural adjustment, acculturation) when these students return to their country-of-origin. Therefore, the purpose of the present study was to extend the current literature by examining both the experiences of adjusting to a host culture and readjusting to a culture-of-origin after returning from a study abroad experience to develop a sustainable education system across cultures.

Socio-cultural adjustment refers to the ability to adapt to a new cultural environment, facilitated by acquiring the necessary skills and knowledge within a social learning framework (Bierwiaczonek and Waldzus, 2016). The acculturation process involves changes in identity, relationships, and worldview about self, others, and environments (Karataş et al., 2020; Berry, 2005). But as opposed to immigrants who need to acculturate to a new country, most international students return to their countries-of-origin at some point. Re-entry is defined as the process of re-adapting to one’s home culture after experiencing cultural adjustment abroad (Sussman, 2002), and the process of understanding changes in the home environment and renegotiating re-entry relationships (Brown and Holloway, 2008). Often, the re-adjustment process can be difficult due to the changes that have occurred in both the environment (e.g., scenery, pop culture, technology) and the persons (e.g., students themselves, people who are left behind in the home country, public) while they were absent and because many people assume that the returners will know how to navigate their home country despite.

Ward et al. (2001) identified affective, behavioral, and cognitive components of cultural shock when sojourners encounter the host culture. The affective component may include confusion, anxiety, bewilderment, and an intense desire to be elsewhere. The behavioral aspect is related with cultural learning, including social skills, learning about rules, conventions, and interpersonal rules. The cognitive element refers to influences in their worldview, such as how they perceive others and themselves. Similar to those entering a host culture, the returners may experience a similar affective, behavioral, and cognitive cultural shock when they return to their country-of-origin (Gaw, 2000). Common issues these returning students may experience include a sense of loss about leaving the host country, the roles with which they have become familiar, the established relationships, and the routines that were formed, as well as losing access to lifestyle or resources that are not available in the home country.

According to the U-curve theory (Oberg, 1960), people who study or work abroad typically experience stages of culture shock until they achieve satisfactory adjustment. The process starts with the Honeymoon stage, characterized by enthusiasm and excitement about the new cultural environment, followed by a crisis stage, characterized by cultural shock, disappointment, distress, and even hostility. Subsequently, people experience a transition stage, characterized by gradual adaptation to the new culture. Finally, people reach the mastery stage with adjustment integration and enjoyment of living in the host country. Gullahorn and Gullahorn (1963) expanded the U-curve model to a W-curve model incorporating the re-entry phenomenon, which repeats the honeymoon stage, crisis stage, transition stage, and mastery stage when they return to their home country. International students who never achieve the transition stage or adjustment stage in their host country might find it more difficult to gain self-efficacy to actively deal with the changes in cultural environment when returning to their country of origin and repeating the same cycle. However, while the ideas offered by Gullahorn and Gullahorn (1963) are interesting, surprisingly little research has been conducted on the experiences of international students when they return to their country-of-origin. Therefore, the present study attempted to address this limitation by examining the validity of Gullahorn and Gullahorn’s W-curve model (Gullahorn and Gullahorn, 1963) with a sample of South Korean adolescents who studied abroad and returned home.

Developmentally, adolescents are in a stage in which they learn more about themselves and start to develop their own identity in relation to the cultural context in which they reside (Kroger, 2007). International students adapt to and internalize new patterns of interacting and communicating within the cultural norms of the foreign country (Smith and Khawaja, 2011). Adolescents might especially be more susceptible to these changes and internalization of new ways of relating with others (Lee and Pistole, 2014). When international students return to their home country, they encounter some changes in their familiar context, such as environmental changes in the neighborhood such as new stores, popular new TV shows, and new technologies in their schools. They also may experience significant differences between the host country and their home country in regards to school norms and the educational system. Also, educational materials learned from study abroad may not be transferable into the new educational context, and returning students may encounter different relational norms in interacting with peers and teachers (Koo et al., 2021). Furthermore, adolescents are also at a developmental stage in which they learn how to relate with others, and begin to understand changes in themselves and in the environment through interactions with others (Crone and Fuligni, 2020). For example, after going through the social adaptation process in the host country, returning international students may encounter changes in familiar relationships, such as friends and family, which can be a source of stress and difficulty (Butcher, 2002). Frequent shifts of context during this critical adolescent developmental stage can cause confusion and impede a healthy process of identity developmental (Newman and Newman, 2020; Smetana et al., 2006).

A growing number of South Korean students have been relocating to foreign countries as teenagers or young children to acquire competency in a foreign language and prepare for entry into foreign universities (Song, 2010). This trend is also influenced by the Korean education system which has become more and more competitive and demanding (Jung, 2020; Kang and Abelmann, 2011); teenagers often want to escape from heavily test-oriented Korean schools (Ly, 2008). When abroad, students typically live alone on campus housing, with host families, or with one of their parents while the other parent continues to work and send money from Korea (Ly, 2008). The split-family living environment may not provide enough support and supervision for students who are transitioning to study in a foreign country. When they return, adolescents may experience changes in living situations and different family dynamics.

In order to understand the returning adolescents’ readjustment process in their subjective context, we conducted a qualitative study. We examined retuning adolescents’ school experiences before leaving, their study abroad experiences, and their re-entry experiences. We also examined the affective, behavioral, and cognitive aspects of their transition. We also examine if the W-curve model is applicable to retuning Korean adolescents. As noted earlier, there is little empirical research examining reentry experiences of international students. Therefore, it is hoped that this study will provide a better understanding of Korean adolescents’ experiences when returning to Korea from studying abroad as a way of providing a sustainable education system for re-entry international student.

## Method

### Participants

A total of 12 adolescents (6 females and 6 males) who returned from studying abroad participated in a 60 ~ 100 min long interview. The participants’ ages ranged from 12-years to 16-years (*M* = 14.9, *SD* = 1.85). Eight students were enrolled in 6th to 11th grade standard schools and four of them were attending an alternative school in Korea. This study was approved by the Institutional Review Board (IRB) of National Youth Counseling Center in Korea, and the researchers took all required safety measures to protect the human participants. Following the approval, the principal investigator (PI) faxed and emailed five elementary and middle schools in Seoul that operate special education programs for returning international students to inquire about their interest in participating. Three elementary and middle schools decided to take part after being contacted and given more details about the interview. The informed consent procedures were approved by all the participating class teachers and parents. The interview began with a statement explaining the purpose of the study, informed consent, estimated interview duration, and information about potential risks. The participants were required to go to the interview location inside the school after finishing their official class activities. All interviews were conducted in a serene atmosphere at class room; a break was provided in the middle of the interview if necessary. Before the interview to help understanding of this study, they were also informed that the interview contents would remain confidential and were provided with contact information for the PI. They were asked for their written consent to participate in the study after receiving additional verbal explanations. To accurately grasp the content from the participants, we observed and took notes of any non-verbal behaviors and expressions if the participant had any particular emotional expressions. We further narrowed the participants to those who studied abroad for 2–5 years because the length of time spent in study abroad can result in qualitatively different experiences. The 12 participants studied abroad in various countries: 2 were in Canada, 2 were in England, 2 were in New Zealand, 3 were in Australia, and 3 were in U.S. (Table 1).

**Table 1:** Participant’s information of studying abroad.

Number of participants	Gender/age (*N* = 12; 6 females, 6 males)	Countries to study abroad	Start and end of time of studying abroad (ranged from 2 to 5 years)	Live with whom abroad, reason of return to Korea
1	Female	Canada	34 month (from K5 ~ K7)	Living in school dorms, Challenges of adjusting to Canadian schools and Loneliness
2	Male	Canada	40 month (from K6 ~ K9)	Parent and Siblings, Father’s deployment to Korea
3	Female	England	36 month (from K7 ~ K9)	Parent and Siblings, Father’s deployment to Korea
4	Male	England	48 month (from K8 ~ K11)	Parent and Siblings, Father’s business issue
5	Female	New Zealand	35 month (from K6 ~ K8)	Living in school dorms, Family financial hardship
6	Male	New Zealand	26 month (from K6 ~ K8)	Local Homestay, Preparation for Korean University
7	Female	Australia	30 month (from K5 ~ K7)	Living in school dorms, Adjusting to life in a new country
8	Male	Australia	26 month (from K4 ~ K6)	Relative’s house, Family financial hardship
9	Male	Australia	28 month (from K6 ~ K8)	Local Homestay, Family financial hardship
10	Female	United State	60 month (from K7 ~ K11)	Parent and Siblings, Father’s deployment to Korea
11	Female	United State	50 month (from K7 ~ K10)	Parent and Siblings, Father’s business issue
12	Male	United State	54 month (from K7 ~ K11)	Parent and Siblings, Father’s deployment to Korea

### Interview protocol

In the pre-interview session, we informed the participants that the interview would be audio-recorded, and also checked their expectations and potential biases about participating in the interview. The interview was conducted by a research team composed of 2 counseling psychologists and two master’s level counselors. All of the researchers had previously engaged in qualitative research projects and received training in qualitative methodology. A set of open-ended questions was developed at the outset of the study to guide the data collection process.

### Data analysis

The interviews were analyzed using phenomenological approach to identify the core aspects of Korean returning adolescents’ unique experiences. Phenomenology explores the direct experience and perception of phenomena by individuals, demanding an understanding of how a person perspective the world within that context (Crotty, 1996). This study adopted Georgi’s (1985) approach of descriptive phenomenology, which emphasizes observing and describing “what is there” as it appears, rather than focusing on hermeneutic interpretation. Georgi’s (1985) methodology of descriptive phenomenology highlights the importance of identifying meaning units and themes, while giving particular attention to the expressions and perspectives of the research participants (Creswell and Poth, 2016; Georgi, 1985). Therefore, in this study, descriptive phenomenology was considered an appropriate research approach to deeply understand the socio-cultural adaptation and re-adaptation experiences of Korean returning adolescents from studying in English-speaking countries, aiming to reveal their lived experiences as vividly as possible. Finally, this study was oriented toward Georgi’s (1985) descriptive phenomenology, with a focus on providing a descriptive account of the participants’ experiences of phenomena.

Data was analyzed three coding phases to conduct descriptive phenomenology; consists of (a) the initial phase, line-by-line coding, (b) the second phase, where line-by-line codes from the first phase are placed in a higher order category, (c) the third phase, theoretical coding, a sophisticated level of coding used to synthesize and integrate the codes that were selected during the second phase and to show how these codes may relate to each other. To demonstrate a specific example of the three-stage coding process in this study, in the initial phase, line-by-line coding was conducted by repeatedly reading the transcribed interview data to identify keywords and meaning units, with the goal of understanding the overall context and flow of the participants’ lived experiences. For example, in this study, codes related to the W-curve model were generated based on the data to reflect the socio-cultural adaptation and re-adaptation experiences of Korean international students, and the generated codes and data were reviewed. In the second phase, based on the line-by-line coding, the identified meaning units were grouped together to highlight the essence of the unique phenomenon of socio-cultural adaptation and re-adaptation experienced by Korean adolescents returning from studying in English-speaking countries. For example, the sub codes in this study were integrated according to the Honeymoon, Crisis, and Recovery stages. In the “Honeymoon stage,” sub-codes such as “Excited about studying abroad and enjoyed having a less restrictive school environment” were categorized. During this process, the study followed an emic approach, striving to maintain the participants’ perspectives and linguistic expressions as they were, while minimizing the researcher’s preconceptions or biases. In the third phase, the classified codes were integrated to better capture the essence of the phenomenon. For example, the sub-codes generated in the second phase were ultimately integrated into the main code “Initial enthusiasm and positive experiences” within the “Honeymoon stage.” The relationship between the codes was analyzed to provide theoretical insights, explaining how they connect and interact with each other. To ensure the objectivity of the coding process, the interviewers reviewed the coded interviews to give comments on the accuracy of the coding. The research team members discussed until we reached a consensus if we find significant discrepancies between coders.

#### Ethics approval statement

All ethical guidelines were followed as required for conducting human research. Informed consent was obtained from all individual participants included in this study.

## Results

The results of the study were mapped onto each stage of the W-curve model, and the returning students’ crisis stage was further specified as interviewees shared more about their current adjustment issues (Table 2).

**Table 2:** Socio-cultural adaptation and re-adaptation experiences of Korean returning adolescents.

Each stage	Main code	Sub-code
Honeymoon stage of studying abroad	Initial enthusiasm and positive experiences	Excited about studying abroad and enjoyed having a less restrictive school environment
Crisis stage: challenges and difficulties abroad	Experiencing language barrier	The subjective impact of communication challenges on personal identity and social integration
	Homesickness, loneliness, and discrimination	Encountering of emotional challenges of longing for connection and belonging though the isolation and facing discrimination
	Relationships with co-national students	Frienamy paradox of co-national student in close-knit communities
	Interactions with family	The impact of familial transition and emotional turmoil
	Experiencing a return crisis as an involuntary transition	Returning because their parents requested it or due to difficulties adjusting to the host country
Recovery stage: adapt and thrive in new environment	Adapt and thrive in new environment	Gaining language competence and working toward an adaptive adjustment to the host culture
Honeymoon stage: preparation for returning	Preparation for returning as a complex emotional experience	Escape to familiar yet unknown situation
Crisis stage back in Korea: difficulties caused by cultural differences when they return	Academic performance	Difficulties of catching up the past
	Teacher relationships	Authority-based teacher relationship and student resistance
	Social rejection upon return	A cultural re-entry phenomenon similar to scent-based exclusion in animals
	Nostalgic idealization of the past	Longing for what was left behind
	National identity crisis	Feeling confused about their national identity
Recovery stage in Korea	Active reentry coping strategies	Actively dealing with readjustment, such as improving closer relationships with others
	Perceived changes in themselves	Mastery of two world

### Honeymoon stage of studying abroad: initial enthusiasm and positive experiences

Students reported they were excited about studying abroad and enjoyed having a less restrictive school environment. Exhilaration of Encounter and more opportunities to participate in extracurricular activities and exercise classes. Similar to their cultural enthusiasm and exploratory optimism, most of them perceived positive experiences with teachers in the host country, reporting that their teachers were very kind and helpful during the transition.

“Teachers spoke slowly and very kindly to me”

“Teachers in the Canada were more like friends, rather than authority figures, and they were very nice to me.”

### Crisis stage: challenges and difficulties abroad

Participants reported that they experienced language barriers, homesickness, discrimination, difficulty adjusting to a different school setting, and difficulty in forming relationships with either Korean or non-Korean students.

#### Experiencing language barrier: the subjective impact of communication challenges on personal identity and social integration

Most of the students reported that they experienced language barriers, the inability to communicate fully to understand others and express themselves that leads to frustration and social withdrawal. One student recalled crying in a school office the first year because he could not understand his English class assignments. Another student explained how his personality changed due to difficulty voicing what he wanted to say in English: “*I could not speak up when I needed to because I could not speak English. That changed my personality.”*

#### Homesickness, loneliness, and discrimination: encountering of emotional challenges of longing for connection and belonging though the isolation and facing discrimination

Some students mentioned that, while they initially appreciated their freedom and lack of parental supervision, they started to miss home. This reflects the emotional struggle of longing for familiar environments and connections, which is important for a sense of belonging. Additionally, students found it challenging to form close relationships with local students in the host country, leading to feelings of isolation and a desire for meaningful connections in a foreign setting. Most students also noted that they had little interaction with local students during their time studying abroad: “*Most of contacts I had were with teachers*.” Students also reported feeling isolated and experiencing discrimination from other students, highlighting the profound effects of perceived bias and exclusion, influencing self-perception and social interactions. A student who studied in the U.S. reported experiences with racial discrimination. Another student who studied in New Zealand stated, “*They called me kiwi. They did not like the smell of my food and told me to go back to Korea because I am smelly and disgusting*.” A student who studied in the Canada reported witnessing unethical behavior in the education system when her science teacher told her that he would pass her if she paid him money.

#### Relationships with co-national students: frienamy paradox of co-national student in close-knit communities

In terms of relationships with other Korean students who were also studying abroad in the host country, students reported that they initially enjoyed befriending them because they felt lonely and had difficulty making friends from other nationalities. However, students also reported feeling isolated and even experiencing bullying even within the Korean international student community, as described below.

My best friend spread out a rumor about a guy I liked and me to other Korean international students, so I did not have anyone talk with me for 3 or 4 months. I felt betrayed and fought a lot with other students. I threw a chair into the desk, and we even got into physical fights. All other students got suspended from the school, and that’s why I had to go back to Korea.

It appears that Korean international student communities in host countries can function as a support but can also be a source of conflict (not sure if this is the right idea) because of the closed and small nature of the community. Additionally, some students reported that other Korean international students provided more opportunities for misbehavior due to less intense supervision and feelings of loneliness. A student said, “*I used to skip classes and go to karaoke with older Korean students who studied abroad here*.”

### Interactions with family: the impact of familial transition and emotional turmoil

Some students reported that they experienced changes in their family’s living situation while studying abroad: One student said, *“At first, the whole family stayed in England together, and then my father left for work in Korea. It was really hard not having my father around*.” Another student related their study abroad experience and return to family situations, including their parents’ divorce. Ongoing family conflict impeded her readjustment process in Korea, with the student revealing, “*My parents got divorced and then my father hit me so badly. So I run away from home and stayed in my friend’s place until my mom took me*.” Some students reported that they did not receive close care or supervision from anyone while studying abroad. “*While I studied abroad for 3 years, my father never came. My mom came several times but she did not say much. She had to come for work, and she just gave me Korean snacks and money. That’s it.*

#### Experiencing a return crisis as an involuntary transition

When it comes to the experience of a return crisis, most students reported that their return might not have been voluntary. They mentioned that they experienced a return crisis because their parents requested it or because they had difficulty adjusting to the host country. Parent-related factors included financial difficulties due to changes in the family situation (e.g., parents’ job-related issues, parents’ marital conflicts/divorce, visa issues). A few students experienced a return crisis because of problems they caused in the school setting and/or in relationships with others (e.g., homestay family), including incidents of violence and disciplinary violations.

### Recovery stage: adapt and thrive in new environment

A few of the participants appeared to reach the third stage of the w-curve model as demonstrated by gaining language competence and working toward an adaptive adjustment to the host culture. The students reported that they became more extroverted and talkative by having more opportunities to participate in discussion and active learning in class.

#### Honeymoon stage: preparation for returning as a complex emotional experience: escape to familiar yet unknown situation

Only a few students reported that they decided to return after taking the time to prepare. This involved discussing potential difficulties with their family and gathering information about the educational requirements in Korea. Others did not engage in much preparation, and some reported that their decision to go back to Korea was abrupt and that they were unprepared for the return. In terms of emotional reactions about returning, most students reported mixed feelings, including excitement about being close to family and friends, worry about adjusting to different levels of educational material (e.g., math, science), and fear of failing in academic performance. However, students who had severe problems in their host country (e.g., being mistreated by other students, failing classes) seemed to be more eager to return home and avoid these problems.

### Crisis stage back in Korea: difficulties caused by cultural differences when they return

Students reported that their excitement about coming back to their home country faded as they experienced difficulty in academic performance, following school rules, conflict with teachers, parents, and peers, and in adjusting to their living environment.

#### Academic performance: difficulties of catching up the past

Returners reported difficulty catching up with different materials that were standard in the Korean education system. Students indicated especially having difficulties in certain areas that were not not taught in their study abroad programs, such as Chinese and written language. Some students reported that they ultimately transferred to alternative schools because they could not keep up with the classes in standard public or private schools. Returners also reported struggling with changes resulting from their overseas experience, including having less freedom and more supervision than they had experienced while studying abroad. Students also complained about having to follow strict regulations in schools, such as regulations concerning acceptable hair styles (e.g., pony tails, keeping hair at a certain length, not being allowed to color hair), banning of ear piercings and wearing accessories, having to wear a certain clothing style or uniform, the size and color of bag they can carry, certain types of indoor shoes in classroom, and having a certain size of bag for indoor shoes. A student mentioned that it was hard for him to no longer have flexibility in his schedule and not being able to skip classes. A student said, “*In New Zealand, if I am coughing, teachers asked me to go home so it will not contaminate other students. In Korea, even when I feel really sick, I have to stay in school and cannot go home.”* Students also reported that they missed taking part in extracurricular activities and exercise classes:, “*In* Australia*, I used to learn musical instruments after school, but now I have to get tutoring for each subjects every day after class.*

#### Teacher relationships: authority-based teacher relationship and student resistance

Students reported that teachers in Korea are stricter and rule-oriented than teachers in the foreign countries where they studied. One student described the differences in teacher relationships, saying, “*In Korea, adults are so proud of themselves. Teachers never to making a mistake or are apologize for something*.” Students also reported that they could not have friendly conversation or engage in small talk with their teachers, saying: “*In English, we do not use different languages to show respect to teachers. Everyone talk like a friend. It was really comfortable. We joked around. In Korea, there is a thick wall between student and teachers.”*

Additionally, students reported that teachers in their host countries were nicer and friendlier. For example, a student said, “*In Canada, teachers do not use physical punishment. If a teacher hits a student, the teacher would be fired, but that’s not the case in Korea. If you did not do homework, so your teacher would rasp your hands*.”

#### Social rejection upon return: a cultural re-entry phenomenon similar to scent-based exclusion in animals

Students reported difficulty making friends and relating with other students when they returned. Some students reported that they were not sure which social group they fit in with and noticed several sub-groups in their classes. Some students also reported that people had biases toward early study abroad, such as assumptions that only rich and spoiled kids study abroad, making it difficult for returners to make friends. A student said, “*People think that I am a rich snobby kid from Gangnam when they found out that I studied abroad*.” Students also reported that since they studied abroad, others expected them to be fluent in English, which became a source of stress. One student said,

People always ask me to speak English. I am so tired of it. Even in math class, the teacher asked me to explain the mathematical formula in English. I even don’t understand that in Korean. How can I explain it in English? Then the teacher told me I am stupid and dumb. I don’t like Koreans to speak so directly.

Some students (6 participants) explained that they did not learn to speak English very well because they usually interacted with other Korean international students in the host country. Some students reported that their English grade was not higher than other Korean students who never studied abroad because the English tests in Korea are heavily grammar oriented, and do not incorporate much listening and speaking. A student said, *“When I did not get a good grade in English class, people made fun of me. People expect me to be fluent in English, and that’s stressful. Teachers call me ‘New Zealand,’ not my name.”* Another student said, *“In writing assignments, in the U.S. teachers focused on the overall ideas or how I think about the topic, but in Korea, teachers only care about grammar in grading…”*

Some students reported that they had difficulties relating to peers in Korea when they returned because they were not up to date with recent popular culture and current trends, although students were able to catch up eventually.

In sum, students appeared to have difficulties in their relationships with peers because people perceived them differently after knowing that they had studied abroad. Students expressed disappointment that they still could not make friends after returning to Korea. Perhaps it can be speculated that this conflict between returning students and their peers might be related to a societal emphasis on maintaining harmony within groups. In a collectivistic society like Korea, being different from others is usually not considered a virtue, and having studied abroad sets students apart from the group when no one else has had a study abroad experience. Language competency and accent changes also were reported as difficulties during the transitioning process, which affected returning students’ relationships with peers.

#### Nostalgic idealization of the past: longing for what was left behind

Students expressed a sense of loss upon leaving the host country including leaving the roles, relationships, routines, and access to lifestyle or resources unavailable in their country of origin. Initially, most students did not consider returning to Korea when they started studying abroad. However, they began to miss various aspects, such as the scenery, people, and culture of the host country. One student said, “*I miss parks and nature in the host county because Seoul is too city*.” A student reported that he continually considers going back to his host country because he missed it so much. Seven students reported that they started recalling their study abroad experience with a more positive perspective after experiencing the Korean education system upon their return and regretted not studying harder while abroad. Some students even idealized their study abroad experiences, although they reported having experienced several difficulties in their host country.

#### National identity crisis

There have been reports of students feeling confused about their national identity. Some have expressed a desire to be a German or American because they believe it is more prestigious than being a Korean. Others have mentioned feeling like they do not belong to any country, with one student even being referred to as an “international orphan” by friends.

### Recovery stage in Korea: active reentry coping strategies

Some students appeared to reach the 3rd stage as demonstrated by reports of feeling comfortable in their surroundings, expectations, and relationships after their return. These students appeared to deal actively with the readjustment. Students reported that they needed to present themselves not as assertively and not differently from other students to be included within their peer social groups. Students emphasized the importance of not openly showing that they had studied abroad, even when others knew about their study abroad experiences, by not mentioning anything about their study abroad experiences, not speaking the language of the country they studied or being careful not to speak with accents; one student stated: I tried to resemble with other students to get closer with them. *To make friends, I tried to say hi loudly and try to approach them first.*

In contrast, students who had had severe levels of difficulties with academic performance and behavioral problems in the foreign country seemed to continue having issues upon returning (e.g., somatization symptoms, self-harm). This may reflect that students’ strategies for coping with changes in the environment remained constant. It may also reflect that these students had limited support from their family during the study abroad experience and at return.

### Perceived changes in themselves: mastery of two world

The worldview of returning international students seemed to have changed because of their experiences. When adapting to a new culture, these students were expected to learn and adopt different social norms and perspectives. As a result, upon returning to their home country, these students experienced changes in their behavior, thinking, and interactions with others. Behaviors that were considered appropriate in the host country became inappropriate upon their return. For instance, a student who had to be talkative and outgoing to make friends in the U.S. reported that they had to learn to be quiet and non-assertive when they returned to Korea. This suggests that when students acquire different ways of behaving based on the cultural context and accept different parts of themselves, they might develop a more integrated sense of self.

## Discussion

This study aimed to enhance teachers and youth counselors’ understanding of the re-entry transition challenges faced by international students, specifically Korean adolescents, upon returning from studying abroad. The results highlighted the complex nature of readjustment, consistent with the W-curve model (Gullahorn and Gullahorn, 1963), which provides a framework for understanding the emotional and psychological phases of cultural adaptation and re-adaptation. Most students in the study did not fully progress through the stages of adaptation while abroad, which potentially disrupted their ability to fully adjust upon returning to South Korea. The results of this study showed that master of two worlds is the stage of the hero’s journey in which the hero can move seamlessly between the two worlds, without destroying or compromising either. Master of two worlds is stage 16 of Joseph Campbell’s hero’s journey, from *The Hero with a Thousand Faces*. These disruptions and incomplete transitions contribute to the challenges observed in their socio-cultural adaptation. Instead of being a Master of two worlds they get “Lost Between Two Worlds.”

The academic landscape, both overseas and in South Korea, posed profound challenges for the participants, particularly in terms of the mismatch between the skills and knowledge acquired abroad and the demands of the Korean education system. Students are involuntarily compelled to confront the familiar yet unfamiliar circumstances that arise as they are required to resume their studies from a point that they have previously neglected while they were overseas. This dissonance between educational frameworks proved to be a critical factor in undermining students’ academic self-efficacy and fostering deep frustration and a sense of helplessness. Many students experienced a tangible decline in their academic performance, which affected on their self-esteem and compounded the emotional toll of these educational hurdles. This underscores the urgent need for schools and educators to be more attuned to the stark differences between systems and to provide the necessary support for students navigating these complex transitions.

It seems that students who had difficulties in school, peer relationships, and/or difficulties with parents (e.g., parents’ disengagement in the children’s issues or too much engagement) before studying abroad continued to experience problems while studying abroad, which impaired the support they received from their parents while in the host country. Students who had to live by themselves without being accompanied by any of their family members exhibited more difficulties and showed a tendency to rely on other international Korean students, and perhaps consequently making them more vulnerable to negative peer pressure and engaging in problematic behaviors in abroad. The result is consistent with previous study indicating how gossip function as a behavioral regulation among international students from the same ethnic background when they study abroad (Lee and Pistole, 2014).

Similar to the adjustment difficulty in study abroad, one of the major challenges for returning students was the difficulty in re-establishing harmonious peer relationships, which is also found in previous studies (Koo et al., 2021; Shu et al., 2020). In the animal world, scent plays a vital role in social cohesion and identity. When an animal returns to its group with an unfamiliar scent—acquired from an external environment—the group may perceive it as a threat, leading to its exclusion or isolation. This exclusion is not because the animal has inherently changed, but because its outward markers of identity have been altered by its time away. Similarly, individuals returning to their home country after studying or working abroad often face a cultural re-entry shock. Upon returning, students reported struggles in understanding social cues, pop culture references, and developing friendships due to their time away. This highlights a critical aspect of the socio-cultural adjustment process, where returning students feel isolated or “different.”

Students who returned to Korea because of problems they exhibited while studying abroad seemed to have more difficulties re-adjusting to Korea. Therefore, these readjustment issues are related to the external factor influencing students before and during study abroad. It also appears that family support is critical in students’ readjustment, which is consistent with previous finding (Shu et al., 2020). Although some parents had the perception that students having difficulty in Korea may adjust better in different contexts, studying abroad does not seem to be an optimal way of escaping the difficult situation. Instead, to help students who are struggling, it could be more effective to build coping strategies and making a collaborative decision about studying abroad based on enhancing future prospects, rather than only to avoid potential problems in Korea (e.g., the competitive and demanding school system in Korea).

Although students reported that they had difficulties when studying abroad, students in crisis stage upon returning to Korea seemed to reflect on their overall experience with a selective memory, focusing mostly on positive experiences, which is consistent with previous study (Gaw, 2000). Many returning international students experience what can be described as a nostalgic idealization of their time abroad, viewing it through a sentimental lens that elevates their experiences, relationships, and even the lifestyle they had in the foreign country. In relation to the school environment and education system, students seemed to especially idealize their foreign schools and perceive their Korean schools as completely opposite from their study abroad education experiences. It would be important to help them understand what they can do in the adjustment process, instead of focusing solely on the different environmental context, in order to have a feeling of control over the current situation and shift to an inward perspective.

Returning adolescents reported difficulty with understanding the different rules, conventions, and assumptions for developing harmonious interpersonal relationships when they returned which can be a part of socio-cultural adjustment process, which is consistent with previous studies emphasizing the role of social support in adjustment process (Shu et al., 2020). Participants in this study reported that they had difficulty understanding the context of their peers’ conversations due to limited knowledge of the current pop culture. Students’ acculturation process to the host country might be different depending on whether they plan to return to Korea or not. Often, international students are advised not to keep too much close contact with their home country’s culture, so that language acquisition can be facilitated (e.g., watching American TV shows instead of Korean shows). However, it seems that maintaining a connection with their home country’s pop culture would be helpful for their re-acculturation if they plan to return. Especially for the students who reported having difficulties with being perceived as different from others because of having studied abroad, a better understanding of Korean culture would lessen the feeling of being “excluded” or being “different” from others.

In contrast to immigrants, international students are expected to learn new roles, while maintaining their traditional roles from their home country (Khan et al., 2015). They need to become master of two worlds. Many cultures tend to resist change, viewing it with caution or negativity (Matsumoto and Hwang, 2020), and can cause conflict when international students return to their home country. This aversion to change explains students’ reports that their strategy for readjustment was focusing on presenting themselves as similar to other students and not displaying behaviors that would differentiate them (i.e., talking about studying abroad, being assertive). Returners may not be aware of internal changes or behavioral changes that they have undergone, and assume that they will fit in with their home country right away. This expectation could make the re-adjustment process more difficult. As the results from this study support the W-curve model, counselors and teachers can help normalize and validate students’ crisis stage as a natural part of the adjustment process.

The results of this study show that there is a need to develop a systematic approach to facilitate returning international students’ readjustment to Korea. There were several students who demonstrated active coping with their readjustment difficulties by seeking support and actively learning and adjusting when they returned. However, more students showed severe levels of distress such as depression, anxiety, anger management issues, self-harm, or violence, and had difficulty expressing their concerns to others. Psycho-educational outreach programs, support groups, and returning preparation programs would assist returners become more aware of changes they experienced abroad and changes they might encounter when they return. Usually returners spent less time preparing for their return, compared to the time they spend preparing for study abroad (e.g., learning languages or learning about different cultural norm). Returning can also be particularly difficult for students who did not return voluntarily. People tend to remain in a familiar locale and with familiar people, especially people who they are attached to Bowlby (1973) and Shaver et al. (2016), and preparing students for losses they might experience while discussing strategies for keeping connections with close friends/teachers via technology (e.g., Facebook) after they return would provide emotional security during the transition.

As the result of the study confirmed the W-curve model that international students repeat their adjustment cycle, it suggests that a sustainable education system for re-entry international students is needed. In other words, the W-curve model highlights the significance of experiencing the entire process of cultural adjustment rather than simply adapting to a host culture. Successful adaptation in one culture should not result in maladjustment when transitioning back to the home country or moving to another cultural context. Recent research emphasizes the importance of intercultural competence as a critical skill for international students, enabling them to navigate different cultural environments flexibly and effectively (Lantz-Deaton and Golubeva, 2020). Programs that promote students’ skills set to adjust to another culture, such as resilience, critical thinking, and intercultural communication skills are essential for preparing students to manage cultural differences and social norms across various educational and social settings (Tripon et al., 2023). Additionally, incorporating reflective intercultural learning opportunities, such as workshops and mentoring programs, allows students to understand and articulate their cultural experiences, which enhances their ability to cope with and thrive in diverse cultural situations. By emphasizing a holistic and sustainable approach to international education, institutions can help students build lasting skills that support their long-term well-being and success across multiple cultural contexts.

## Conclusion

The findings of this study reveal that socio-cultural adaptation is central to the readjustment process for South Korean adolescents returning from studying abroad. Through the lens of the W-curve model, which outlines phases of adjustment (honeymoon, crisis, and recovery), the study highlights not only the academic difficulties students face but also the critical socio-cultural challenges that arise when reintegrating into their home culture. These challenges underscore the importance of developing more holistic and integrated support systems for returning international students.

The findings suggest that socio-cultural adaptation, or the ability to re-navigate and re-negotiate social relationships and cultural norms, is often one of the most difficult aspects of reentry for these students. While abroad, students often develop more outgoing or independent personalities in response to the different cultural norms in their host countries. Upon returning, however, many report that these new behaviors become sources of tension within their home culture, which often values conformity and adherence to established social norms. This aligns with the previous study emphasizing multicultural personality as a critical factor in international students’ socio-cultural adaptation (Lee and Ciftci, 2014). As Tripon et al. (2023) suggest, creating educational spaces that encourage intercultural dialog and diversity can help returnees feel more included and understood. Schools and universities should foster an environment where returnees’ international experiences are viewed as assets, not liabilities. By celebrating diversity and promoting intercultural understanding, institutions can help returnees navigate the socio-cultural adaptation process more effectively.

By understanding the W-curve model and its implications for socio-cultural dynamics, educational institutions can take a more active role in supporting returning students. As highlighted by Tripon et al. (2023), fostering educational environments that promote well-being and cultural sustainability is essential. By incorporating the strengths and experiences of returning students into the curriculum, schools can create more inclusive, adaptable, and globally minded communities that benefit all students.

## Limitations

The sample size of this study was small and was limited to students referred by their parents or school administrative for having troubles in school. Therefore, the samples in this study might only represent students who experience more problems generally than other returning students. Thus the generalizability of the results of this study to other returning students could be limited. Also, the country of study abroad and the duration of study abroad were varied in this sample. The sample was also collected only in Korea; international students from other countries might have different experiences when they return to their home country.

## Data Availability

The raw data supporting the conclusions of this article will be made available by the authors, without undue reservation.
